# The Use of Machine Learning to Create a Risk Score to Predict Survival in Patients with Hepatocellular Carcinoma: A TCGA Cohort Analysis

**DOI:** 10.1155/2021/5212953

**Published:** 2021-11-30

**Authors:** Samer Tohme, Hamza O Yazdani, Amaan Rahman, Sanah Handu, Sidrah Khan, Tanner Wilson, David A Geller, Richard L Simmons, Michele Molinari, Christof Kaltenmeier

**Affiliations:** Department of Surgery, University of Pittsburgh, Pittsburgh, PA, USA

## Abstract

**Introduction:**

Hepatocellular carcinoma (HCC) accounts for approximately 90% of primary liver malignancies and is currently the fourth most common cause of cancer-related death worldwide. Due to varying underlying etiologies, the prognosis of HCC differs greatly among patients. It is important to develop ways to help stratify patients upon initial diagnosis to provide optimal treatment modalities and follow-up plans. The current study uses Artificial Neural Network (ANN) and Classification Tree Analysis (CTA) to create a gene signature score that can help predict survival in patients with HCC.

**Methods:**

The Cancer Genome Atlas (TCGA-LIHC) was analyzed for differentially expressed genes. Clinicopathological data were obtained from cBioPortal. ANN analysis of the 75 most significant genes predicting disease-free survival (DFS) was performed. Next, CTA results were used for creation of the scoring system. Cox regression was performed to identify the prognostic value of the scoring system.

**Results:**

363 patients diagnosed with HCC were analyzed in this study. ANN provided 15 genes with normalized importance >50%. CTA resulted in a set of three genes (NRM, STAG3, and SNHG20). Patients were then divided in to 4 groups based on the CTA tree cutoff values. The Kaplan–Meier analysis showed significantly reduced DFS in groups 1, 2, and 3 (median DFS: 29.7 months, 16.1 months, and 11.7 months, *p* < 0.01) compared to group 0 (median not reached). Similar results were observed when overall survival (OS) was analyzed. On multivariate Cox regression, higher scores were associated with significantly shorter DFS (1 point: HR 2.57 (1.38–4.80), 2 points: 3.91 (2.11–7.24), and 3 points: 5.09 (2.70–9.58), *p* < 0.01).

**Conclusion:**

Long-term outcomes of patients with HCC can be predicted using a simplified scoring system based on tumor mRNA gene expression levels. This tool could assist clinicians and researchers in identifying patients at increased risks for recurrence to tailor specific treatment and follow-up strategies for individual patients.

## 1. Introduction

Hepatocellular carcinoma (HCC) is the most common primary tumor of the liver and a leading cause of cancer death worldwide [1]. Within the USA, nearly 42,230 new cases and 30,230 estimated deaths of HCC will occur in 2021 [2]. Despite recent advances in therapeutic intervention, such as liver transplantation, surgical resection, locoregional therapies, and chemotherapy, the recurrence and overall survival rates remain poor [3]. Patients with localized HCC usually have 5-year OS rates of 30%, and they are less than 5% for patients with distant metastasis [4]. Etiologic factors including underlying liver disease as well as stage of presentation greatly vary between patients. In addition, intra-tumoral heterogeneity influences the ability to predict outcomes as well as develop individualized therapeutic strategies for patients [5].

Over the last decade, numerous attempts have been made to find biomarkers that can detect HCC in early stages, help predict disease-free survival (DFS) and overall survival (OS), and establish guidelines for long-term prognosis of HCC [6]. Traditional serum markers, particularly alpha-fetoprotein (AFP) and AFP mRNA, have been found to be prognostic [7]. However, they rely on significant tumor burden and often have poor sensitivity and specificity in relation to the cutoff value used; taking this into consideration, their usefulness is often questionable [8].

Recent years have shown a rapid development of predictive biomarkers with advances in the understanding of tumor biology and the use of data mining through bioinformatics. A large series of studies has described the role of tissue and serum markers, oncogenes, tumor suppressor genes, and microRNAs in HCC prognosis [9–11]. However, the majority of scoring systems that have been developed are often complicated and impractical in the real-world setting [11, 12].

The aim of the current study was to create an easy-to-calculate gene-based risk score using machine learning to predict outcomes in patients with hepatocellular carcinoma using the Cancer Genome Atlas (TCGA) public database. Here, we found that the developed risk score was able to stratify patients into different risk groups for shorter DFS and OS.

In general, such models should not be viewed as replacements for good clinical judgment but as additional instruments to assist clinicians in counseling and choosing individualized treatment strategies for every patient.

## 2. Methods

RNA-Seq and corresponding clinical data for liver hepatocellular carcinoma (LIHC) were obtained from TCGA database [13]. A list of 363 samples was obtained. We used GEPIA: a web server for cancer and normal gene expression profiling, to determine the top 75 genes with highest impact on DFS [14]. CBio Portal was used to extract mRNA gene expression and sociodemogrpahic data as well as clinical characteristics [15]. Clinicopathological data of the study population were limited to age, sex, ethnicity, tumor stage, and histologic grade.

We next performed ANN analysis to determine the relative weight of the chosen genes and their impact on DFS. For this purpose, a 10-fold cross validation methodology was used, in which the whole dataset was randomly divided and 90% of the patients were selected for the training step and 10% were selected for the final testing. The final model was the one that maximized the correct classification of patients by DFS outcomes. The importance of independent predictors represented a measure of how much the predicted values changed with variations of the independent variables. Genes with a normalized importance >50% were used for subsequent CTA. CTA did not require assumptions on the distribution of variables or linearity of the data and could handle highly skewed or multimodal continuous variables [16]. The output of CTA provided cutoff values for the top three genes predicting DFS. We then used a simple scoring system (0 or 1 point) to give points for each gene based on the individual gene cutoff levels that were derived through CTA. Patients were then grouped based on their total scores (0–3). DFS and OS were obtained by Kaplan–Meier survival analyses (log-rank test). We furthermore examined the association for RFS and OS of the new scoring system and multiple other variables using Cox proportional hazard regression analysis.

## 3. Results

### 3.1. Neural Network Analysis and Classification Tree Analysis

**Figure 1:**
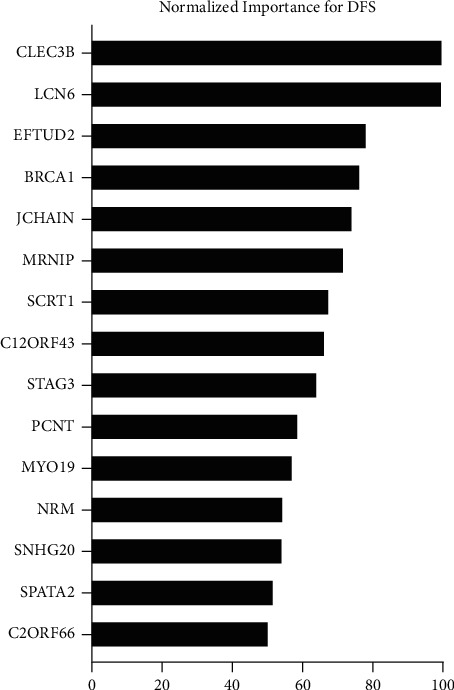
Artificial Neural Network (ANN) analysis of the 75 most significant genes predicting DFS was performed. Shown are the 15 genes along with their adjusted normalized importance.

**Figure 2:**
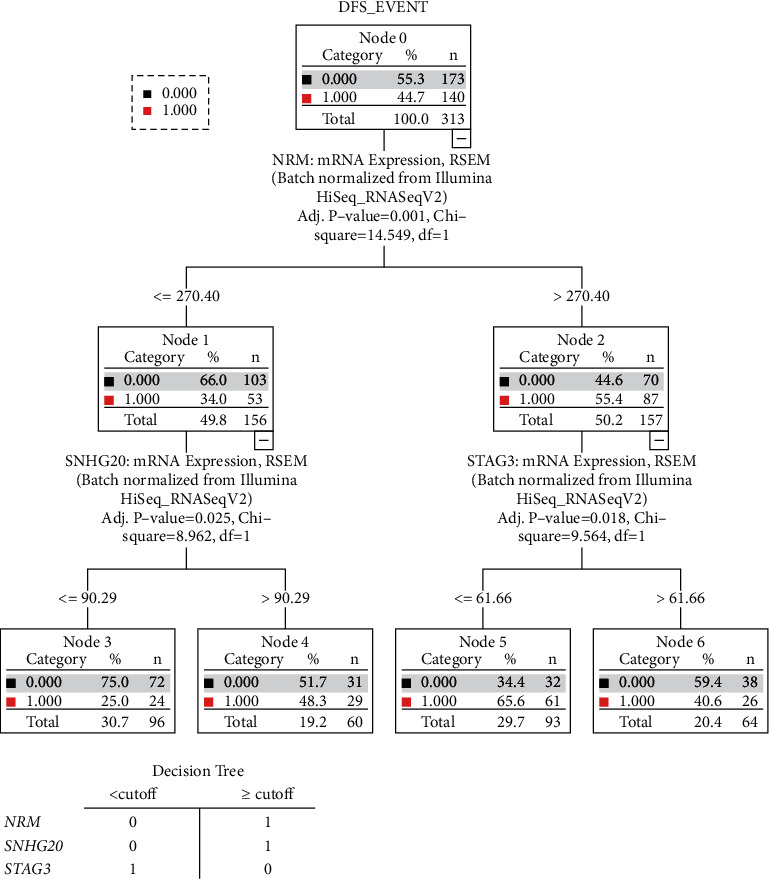
(a) Classification Tree Analysis was performed with the 15 most important genes derived from NNA. CTA provided three genes that were most predictable of survival along with cutoff values. (b) Based on the individual cutoff values and their predictability for survival or death, a score of either 0 (higher percentage of survival) or 1 (higher percentage of death) was given.

A total of 363 patients with biopsy-proven HCC were derived from TCGA-LIHC database. The 75 most significant genes that predict DFS along with normalized mRNA expression levels were derived from GEPIA and cBioPortal. ANN identified 15 genes with normalized importance > 50% (Figure 1). We next used these 15 genes to perform CTA. Here, we identified Nuclear Envelope Membrane Protein (NRM (Ensembl: ENSG00000137404)), Stromal Antigen 3 (STAG3 (Ensembl: ENSG0000066923)), and Small Nucleolar RNA Host Gene 20 (SNHG20 (Ensembl: ENSG00000234912)) as the strongest independent predictors of DFS. Detailed CTA along with node cutoff values for each gene can be obtained from Figure 2.

### 3.2. Score Development

**Figure 3:**
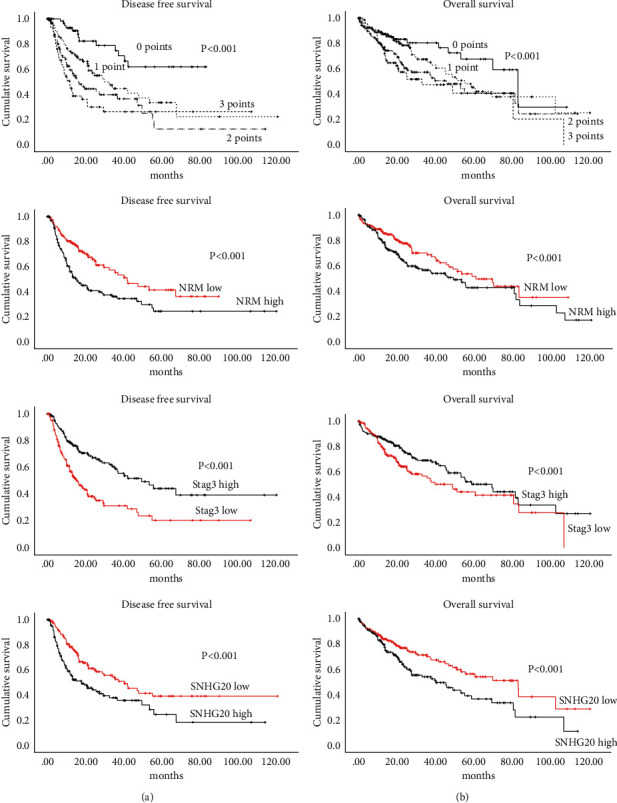
(a) Disease-free survival and (b) overall survival: Kaplan–Meier curve of calculated scores and Kaplan–Meier survival curves for each gene based on cutoff value derived from CTA analysis (gene expression < cutoff or ≥ cutoff, respectively) and Kaplan–Meier curve combination of calculated scores.

Following the initial steps of NNA and CTA, we performed survival analysis for each gene. CTA provided node cutoff values, and based on these numbers, we divided the population in below and above the cutoff (Figure 2). Survival analysis showed that patients with mRNA expression levels for NRM and SNHG20 above the CTA cutoff had significantly worse DFS and OS (*p* < 0.01), whereas patients with STAG3 mRNA levels above the cutoff had significantly better DFS and OS (*p* < 0.01) (A and C in Figures 3(a) and 3(b)).

Based on the prediction of survival for each gene, we developed a simple risk score. Patients with NRM and SNHG20 mRNA levels above the cutoff (prediction of worse survival) received 1 point. Patients with STAG3 mRNA levels below the cutoff (prediction of worse survival) received 1 point. A simplified table with scores for individual gene levels can be obtained from Figure 2. Patients were then grouped based on their overall score into 0–3 points.

### 3.3. Patient Demographics and Kaplan–Meier Survival Analysis

Patient demographics for the entire cohort and each scoring group can be obtained from Table 1. Among the 363 patients, the majority was male (*n* = 244, 67.2%) with a mean age of 60 ± 13 years. The majority of patients were White (*n* = 177, 48.80%) with Stage 1 disease (*n* = 167, 48.7%) and had alcohol as underlying risk factor (*n* = 108, 29.8%) and histologic grade 2 (*n* = 169, 47.2%). The distribution for each score was 20.1% (0 points), 30.9% (1 point), 28.4% (2 points), and 20.7% (3 points). There were no significant differences in regard to baseline demographic parameters among the groups. The Kaplan–Meier survival analysis showed that the developed scoring system could stratify among patients for DFS (0 points, median DFS: not reached; 1 point, median DFS: 29.7 months; 2 points, median DFS: 16.1 months; 3 points, median DFS: 11.7 months, *p* < 0.01). Similarly, the same score was able to stratify patients for decreased OS. This information can be derived from Figures 3(a) and 3(b) (overall score comparison (A), NRM (B), STAG3 (C), and SNHG20 (D)).

### 3.4. Predictors of DFS and OS

We next performed Cox regression analysis and found that on univariate analysis, patients with higher scores had significantly worse DFS (1 point, HR (95% CI): 2.57 (1.38–4.80), 2 points, HR (95% CI): 3.91 (2.11–7.24), and 3 points, HR (95% CI): 5.09 (2.70–9.58), *p* < 0.01). On multivariate analysis, patients with higher scores as well as male patients (HR (95% CI): 1.81 (1.21–2.70), *p* < 0.01) did significantly worse. Black (HR (95% CI): 0.16 (0.04–0.67), *p* < 0.01) and Asian (HR (95% CI): 0.49 (0.32–0.74), *p* < 0.01) patients had prolonged DFS. On univariate analysis for OS, patients with higher scores (2 points, HR (95% CI): 2.05 (1.34–4.39), and 3 points, HR (95% CI): 2.42 (1.34–4.39), *p* < 0.01) and advanced stage (Stage 2 HR (95% CI): 2.00 (1.30–3.07), *p* < 0.01) performed significantly worse. This information can be obtained from Table 2.

## 4. Discussion

The significant increase in mortality rates from primary hepatobiliary cancers, particularly over the past decade, has coincided with a rapidly growing interest to seek effective biomarker-driven approaches to determine prognosis and risk of death in patients undergoing treatment [17].

Estimating the individual patients risk of recurrence or death following tissue diagnosis is helpful for physicians and patients. With a certain estimation on long-term prognosis, physicians can better tailor follow-up and patients have the opportunity to make decisions in regard to treatment options and future care. It is therefore of high importance to develop diagnostic tools that are readily available to predict DFS and OS in patients that were diagnosed with hepatic malignancies.

In this current study, we used Neural Network and Decision Tree Analysis to create a genetic signature score to aid in prediction of DFS and OS in patients with HCC. Using the above techniques, we found that the tumor expression levels of STAG3, SNHG20, and NRM significantly differed among patients. With the help of CTA, we transformed the gene expression levels into a scoring system which provided the ability to adequately stratify between patients with different risk for shorter DFS and OS (0–3 points). The calculated scoring system remained a significant predictor for shorter DFS and OS following multivariable Cox regression adjustment.

Given the vast differences among patients and the inherent molecular heterogeneity of the disease and cancer genetics, personalized medicine in cancer can be particularly effective [18]. Recent studies have shown the use of cancer genomic analysis to discover biomarkers for drug sensitivity, drug resistance, and predictors of outcomes along with establishing personalized oncology by targeting HER2-positive patients in breast cancer [19, 20]. It is worth noting that several prior studies have evaluated the importance of the genes that we used in developing this score [21–25]. STAG3 is a subunit of the cohesin complex that regulates the cohesion of sister chromatids during cell division. It has been found to be important in DNA repair, meiosis, and its work as a tumor suppressor gene. The loss of STAG3 has been associated with increased metastasis and drug resistance in melanoma [25, 26]. Similarly, NRM has been shown to play critical roles in chromatin organization, gene regulation, and signal transduction. NRM serves as a scaffold for numerous transcription factors and regulator of transcription and cell division. Its presence and prognosis in cancers have been less frequently evaluated; however, some studies suggest that the upregulation of NRM leads to decreased apoptosis along with enhanced cell migration and advanced cancer stage [23, 24]. Lastly, SNHG20 has been shown to directly predict poor prognosis in HCC patients. High SNHG20 expression can be detected within the tumor but not the healthy background. The SNHG20/EZH2/E-cadherin pathway was also identified as the potential mechanism in promoting tumor progression and epithelial-mesenchymal transition [22].

As with all retrospective studies, there are several limitations associated with this analysis. First and foremost, TCGA cohort analysis provides data from untreated tumors. As a result, any genomic change that happens due to intervention is unaccounted for from a genetic standpoint. In addition, only limited clinicopathologic data are available with a lack of information on tumor size, lymphovascular invasion, resection margin, etc. Within the US, studies have shown that Asians have the highest incidence for HCC followed by Blacks, Hispanics, and non-Hispanic Whites. TCGA-LIHC database underrepresents Black patients. This makes the finding of the study less generalizable and will therefore need to be confirmed in a cohort that is more representative of the current HCC population within the USA [27]. Furthermore, the calculated risk score was created using Neural Network analysis using an intrinsic training and testing cohort. An extended retrospective study to validate the score is currently underway at our institution.

## 5. Conclusion

The current study used individual patient tumor genomic data to develop a three-gene predictive score to stratify patients and their risk for shorter DFS and OS. This study serves to deepen our understanding of how a patient's individual genetic profile can be utilized to better understand their prognosis and consequently improve and individualize their treatment.

## Figures and Tables

**Table 1:** Comparison of demographics by risk score.

	All patients		0 points		1 point		2 points		3 points		*p* value
Gender											0.026
Female	119	32.80%	15	20.50%	37	33.00%	34	33.00%	33	44.00%	
Male	244	67.20%	58	79.50%	75	67.00%	69	67.00%	42	56.00%	
Age, mean STD	60	13	60	13	61	12	60	13	56	14	n.s.
Race											n.s.
White	177	48.80%	43	58.90%	56	50.00%	44	42.70%	34	45.30%	
Black	17	4.70%	3	4.10%	6	5.40%	5	4.90%	3	4.00%	
Asian	157	43.30%	23	31.50%	47	42.00%	52	50.50%	35	46.70%	
Unknown/other	12	3.30%	4	5.50%	3	2.70%	2	1.90%	3	4.00%	
Etiology											n.s.
No risk factor	88	24.20%	13	17.80%	26	23.20%	30	29.10%	19	25.30%	
Alcohol	108	29.80%	30	41.10%	30	26.80%	25	24.30%	23	30.70%	
HBV	74	20.40%	13	17.80%	27	24.10%	18	17.50%	16	21.30%	
HCV	34	9.40%	4	5.50%	7	6.30%	13	12.60%	10	13.30%	
NAFLD	18	5.00%	5	6.80%	7	6.30%	3	2.90%	3	4.00%	
Other/unknown	41	11.30%	1	1.40%	6	5.40%	5	5.00%	1	1.40%	
Stage											n.s.
1	167	48.70%	34	50.00%	61	58.10%	44	44.90%	28	38.90%	
2	85	24.80%	16	23.50%	20	19.00%	24	24.50%	25	34.70%	
3	84	24.50%	16	23.50%	23	21.90%	28	28.60%	17	23.60%	
4	7	2.00%	2	2.90%	1	1.00%	2	2.00%	2	2.80%	
Histologic grade											n.s.
1	52	14.50%	8	11.10%	13	11.70%	22	21.80%	9	12.20%	
2	169	47.20%	45	62.50%	45	40.50%	45	44.60%	34	45.90%	
3	124	34.60%	18	25.00%	47	42.30%	29	28.70%	30	40.50%	
4	13	3.60%	1	1.40%	6	5.40%	5	5.00%	1	1.40%	

**Table 2:** Cox regression: DFS and OS.

	Disease-free survival								Overall survival							
	Univariate analysis				Multivariate analysis				Univariate analysis				Multivariate analysis			
	HR	95% CI		*p* value	HR	95% CI		*p* value	HR	95% CI		*p* value	HR	95% CI		*p* value
Score				<0.01				<0.01				0.010				
0	1 : 00 reference				1 : 00 reference				1 : 00 reference				1 : 00 reference			0.01
1	2.57	1.38	4.80	0.01	3.41	1.76	6.61	<0.01	1.48	0.83	2.62	0.18	1.95	1.03	3.67	<0.05
2	3.91	2.11	7.24	<0.01	5.54	2.87	10.67	<0.01	2.05	1.17	3.60	<0.01	2.46	1.30	4.63	<0.01
3	5.09	2.70	9.58	<0.01	6.36	3.24	12.49	<0.01	2.42	1.34	4.39	<0.01	2.92	1.52	5.62	<0.01
Gender																
Female	1 : 00 reference				1 : 00 reference				1 : 00 reference				1 : 00 reference			
Male	1.13	0.79	1.61	0.51	1.81	1.21	2.70	<0.01	0.81	0.57	1.15	0.24	1.05	0.69	1.59	0.82
Age, mean STD	1.00	0.98	1.01	0.73	0.99	0.98	1.00	0.15	1.01	1.00	1.03	0.07	1.00	0.99	1.02	0.74
Race				0.12				<0.01				0.09				0.13
White	1 : 00 reference				1 : 00 reference				1 : 00 reference				1 : 00 reference			
Black	0.25	0.06	1.02	0.05	0.16	0.04	0.67	0.01	1.31	0.60	2.85	0.50	1.19	0.50	2.81	0.69
Asian	0.76	0.54	1.07	0.11	0.49	0.32	0.74	0.01	0.66	0.45	0.97	0.04	0.67	0.42	1.06	0.09
Unknown/other	0.99	0.36	2.70	0.98	0.63	0.22	1.79	0.39	1.34	0.54	3.32	0.53	1.75	0.69	4.44	0.24
Stage				0.95				0.85				<0.01				<0.01
1	1 : 00 reference				1 : 00 reference				1 : 00 reference				1 : 00 reference			
2	1.03	0.68	1.55	0.89	1.03	0.67	1.59	0.89	1.15	0.73	1.83	0.55	1.10	0.68	1.79	0.70
3	1.14	0.74	1.74	0.56	1.22	0.78	1.90	0.39	2.00	1.30	3.07	<0.01	2.08	1.32	3.28	<0.01
4	1.13	0.28	4.63	0.87	0.97	0.23	4.16	0.97	1.67	0.40	6.91	0.48	2.04	0.48	8.61	0.33
Histologic grade				0.53				0.12				0.43				0.67
1	1 : 00 reference				1 : 00 reference				1 : 00 reference				1 : 00 reference			
2	1.47	0.84	2.58	0.17	1.97	1.09	3.55	0.03	1.07	0.63	1.81	0.80	1.19	0.68	2.09	0.54
3	1.27	0.71	2.26	0.43	1.47	0.80	2.71	0.22	0.94	0.54	1.63	0.81	1.05	0.59	1.88	0.87
4	1.13	0.44	2.93	0.79	1.46	0.50	4.28	0.49	0.34	0.08	1.49	0.15	0.53	0.12	2.41	0.41

## Data Availability

The results published or shown here are in whole or part based upon data generated by TCGA Research Network: https://www.cancer.gov/tcga.
